# Investigation of Herb-Drug Interactions between *Xylopia aethiopica*, Its Principal Constituent Xylopic Acid, and Antidepressants

**DOI:** 10.1155/2024/9923801

**Published:** 2024-05-25

**Authors:** Christian C. Ndu, Wonder K. M. Abotsi, Priscilla K. Mante

**Affiliations:** Department of Pharmacology, Kwame Nkrumah University of Science and Technology, Kumasi, Ghana

## Abstract

**Introduction:**

Depression affects an estimated 350 million people worldwide and is implicated in up to 60% of suicides. Only about 60–70% of patients respond to antidepressant therapy. One of the factors causing patients to not attain therapeutic goals is herb-drug interactions.

**Objective:**

To investigate any potential herb-drug interaction that might exist between *Xylopia aethiopica* extract (XAE) or xylopic acid (XA) and selected conventional antidepressants (imipramine, fluoxetine, and venlafaxine) in mice.

**Methods:**

Dried, powdered fruits of *Xylopia aethiopica* were cold macerated in 70% ethanol to obtain XAE. XA was isolated by cold macerating dried fruits of *Xylopia aethiopica* in petroleum ether, crystallising impure XA with ethyl acetate, and purifying XA crystals with 96% ethanol. Pharmacodynamic interaction was assessed via isobolographic analysis of tail suspension tests of the agents individually and in their respective combinations. Pharmacokinetic interaction was assessed by monitoring the effect of coadministrations on the plasma concentration of antidepressants and xylopic acid via HPLC analysis.

**Results:**

XAE and XA in mice showed significant antidepressant-like activity in the tail suspension test. With interaction indices less than one, synergism of antidepressant effect was observed in the *Xylopia aethiopica* extract/fluoxetine (*γ*_XAE/FL_ = 0.502), *Xylopia aethiopica* extract/imipramine (*γ*_XAE/IP_ = 0.322), *Xylopia aethiopica* extract/venlafaxine (*γ*_XAE/VL_ = 0.601), xylopic acid/imipramine (*γ*_XA/IP_ = 0.556), xylopic acid/venlafaxine (*γ*_XA/VL_ = 0.451), and xylopic acid/fluoxetine (*γ*_XA/FL_ = 0.298) combinations, which may be potentially due to elevation of serotonergic neurotransmission via varying mechanisms. The AUC of imipramine (AUC_IP_ = 1966 ± 58.98 *µ*g/ml.h) was significantly (*P* < 0.0001) reduced by *Xylopia aethiopica* extract (AUC_IP_ = 1228 ± 67.40 *µ*g/ml.h) and xylopic acid (AUC_IP_ = 1250 ± 55.95 *µ*g/ml.h), while the AUC of xylopic acid (AUC_XA_ = 968.10 ± 61.22 *µ*g/ml.h) was significantly (*P* < 0.0001) reduced by venlafaxine (AUC_XA_ = 285.90 ± 51.92 *µ*g/ml.h) and fluoxetine (AUC_XA_ = 510.60 ± 44.74 *µ*g/ml.h), possibly due to the effect of interfering agents on gastric emptying hence reducing oral absorption.

**Conclusion:**

*Xylopia aethiopica* extract and xylopic acid interacted synergistically with imipramine, fluoxetine, and venlafaxine and reduced the systemic circulation of imipramine.

## 1. Introduction

Depression is a mental disorder characterised primarily by anhedonia and dysphoria, among a host of other symptoms, and affects over 350 million people worldwide [1, 2]. Depression is a major risk factor for suicide, accounting for as much as 60% of suicidal cases [2–7]. The national average prevalence of suicide in Ghana has been estimated at 3.3% of the population [8, 9].

Previous research has highlighted that depression may often go unnoticed or untreated, and hence, a significant number of adults experiencing depression do not receive the necessary treatment for their symptoms [10]. When diagnosed, treatment of depression conventionally employs the use of antidepressants, which have been proven to be effective agents for resolving depression. The major classes of antidepressants frequently used include selective serotonin reuptake inhibitors (SSRIs), serotonin-noradrenaline reuptake inhibitors (SNRIs), and tricyclic antidepressants (TCAs) [11].

Of the few depression-diagnosed patients who receive treatment, as much as 30–40% do not attain therapeutic goals [12]. Antidepressant treatment failure can be due to a heterogeneity of factors. One possible factor responsible for this resistance to antidepressant therapy is the occurrence of drug interactions due to the concomitant administration of other drugs and herbs together with conventional antidepressants [12]. With 60% of the global population using herbal medicine, the risk of herb-drug interactions is very high [13–17]. Clinically, caution is advised in herb-neuropsychiatric drug coadministrations as they have been revealed to cause harmful side effects and complications through pharmacodynamic and pharmacokinetic interactions [18, 19]. A herb with a potentially high risk of interacting with antidepressants is the widely used *Xylopia aethiopica*, as its hydroethanolic extract and isolate, xylopic acid, have been discovered to possess antidepressant-like activities via mechanisms similar to those of classical antidepressants [3, 11, 20]. It is therefore critical to determine whether or not this extract or isolate of *Xylopia aethiopica* interferes with the antidepressant effect of orthodox antidepressants.

This study therefore sought to investigate any potential pharmacological interaction that might exist among the hydroethanolic extract of *Xylopia aethiopica* or its major constituent, xylopic acid, and selected conventional antidepressants. The study specifically evaluates the impact on the antidepressant efficacy of imipramine, fluoxetine, and venlafaxine during concurrent administration of XAE or XA, aiming to elucidate potential interactions and their implications for therapeutic outcomes. The central research question guiding this study revolves around determining the extent to which the hydroethanolic extract or isolate of *Xylopia aethiopica* interferes with the antidepressant effects of conventional antidepressants.

## 2. Materials and Method

### 2.1. Animals

Institute of Cancer Research (ICR) mice (26 ± 10 g) were obtained from Noguchi Memorial Institute for Medical Research, University of Ghana, Accra. The mice were housed in the *vivarium* of the Department of Pharmacology, Kwame Nkrumah University of Science and Technology (KNUST), Kumasi, to acclimatise to the laboratory conditions. The animals were housed 10 mice per stainless steel cage, with softwood shavings as bedding, and fed with a commercially available pellet diet, and given water *ad libitum*. Experiments were conducted in accordance with internationally accepted principles for laboratory animal use and care, and ethical approval was obtained from the Animal Research Ethics Committee of KNUST (KNUST 0039).

The sample size of seven animals for this pharmacological study was determined through a meticulous power analysis, ensuring a balance between statistical robustness and ethical considerations. Our power analysis indicated that with this sample size, we could achieve a statistically meaningful level of power to detect expected effect sizes while minimising the likelihood of type II errors. Anticipated effect sizes, drawn from preliminary studies and historical data, validated the feasibility of detecting these effects within the chosen sample. Upholding ethical guidelines advocating for minimal animal use, we selected a sample size that aligned with scientific integrity without unnecessary animal burden. The simplicity of our experimental design allowed for a focused assessment, optimising the sample size to efficiently capture variations and trends. Contingencies for potential attrition and unforeseen circumstances were accounted for, ensuring the reliability of our statistical analyses.

### 2.2. Drugs

The antidepressants (imipramine (IP), venlafaxine (VL), and fluoxetine (FL)) were selected as representatives of the three major classes of antidepressants that are currently used in clinical practice. Imipramine is a tricyclic antidepressant (TCA); venlafaxine, a serotonin-noradrenaline reuptake inhibitor (SNRI); and fluoxetine, a selective serotonin reuptake inhibitor (SSRI). Doses of antidepressants used were selected from literature [3, 20]. They served as standard antidepressants for the validation of the model.

### 2.3. Extract Preparation

Dried fruits of *Xylopia aethiopica* were obtained from Kwahu Asakraka (6°37′45″N 0°41′11″W) in the Eastern Region of Ghana. The fruits were authenticated at the Department of Pharmacognosy, Faculty of Pharmacy and Pharmaceutical Sciences, KNUST. A voucher specimen (KNUST/HM1/2015/FR001) was deposited at the herbarium of the faculty. The fruits were coarsely milled, and 2.5 kg was cold macerated in 70% (w/v) ethanol for 72 h. The extract obtained was then concentrated into a dark-brown, oily sludge (yield 13.34% w/v). The extract, XAE, was subsequently used in the experiments at selected doses 30, 100, or 300 mgkg^−1^. Dosing was based on preliminary toxicity studies, which are not reported in this current study. An HPLC fingerprint of the extract was obtained as described earlier [3, 6]. The column employed was Phenomenex Luna 5*µ* C8 (2) 150 × 4.60 mm. The mobile phase contained water (10%) and methanol (90%). The eluent was monitored at 206 nm.

### 2.4. Xylopic Acid Isolation

Xylopic acid (XA) was isolated as described by Osafo et al. [6]. A mass of 2.5 kg of powdered dried fruits of *Xylopia aethiopica* was cold macerated in petroleum ether for 72 h. The extract obtained was filtered and concentrated via rotary evaporation at 45°C. Xylopic acid crystals were precipitated from the concentrate by the addition of ethyl acetate, and the crystals obtained were purified via recrystallisation with 96% ethanol. A yield of 0.16% (w/w) was obtained. The purity of the isolated xylopic acid was determined by melting point determination and HPLC. The column employed was Phenomenex Luna 5*µ* C8(2) 150 × 4.60 mm. The mobile phase contained water (10%) and methanol (90%). The eluent was monitored at 206 nm. The dosing of the isolate was based on preliminary toxicity studies, which are not reported in this current study.

### 2.5. Tail Suspension Test

The protocol used here was as described by Biney et al. [3]. Randomly grouped mice (*n* = 7) received oral administrations of XA (3, 10, or 10 mgkg^−1^), XAE (30, 100, or 300 mgkg^−1^), fluoxetine (3, 10, or 30 mgkg^−1^), imipramine (3, 10, or 30 mgkg^−1^), venlafaxine (3, 10, or 30 mgkg^−1^), or vehicle (10 mLkg^−1^). The vehicle served as the negative control. At the time of maximum effect (1 h), the mice were individually suspended by their tail, 1 cm from the tip, with an adhesive tape on a horizontal suspension bar that was elevated 52 cm from the base. The duration of escape-oriented behaviours and immobility were recorded with a video camera for 6 minutes and quantified with JWatcher™ by an experienced observer blinded to all treatment groups. Mice that climbed on their tails were gently pulled down, and the test was continued. Scored behaviour was defined as mobility (struggling) and immobility (lack of movement).

This protocol was again employed in randomly grouped mice (*n* = 7) treated with coadministration of extract/antidepressants and XA/antidepressants.

### 2.6. Isobolographic Analysis

The isobolographic analysis employed was similarly described by Woode et al. and Boakye-Gyasi et al. [4, 5]. The ED_50_s of XAE, XA, imipramine, venlafaxine, and fluoxetine were determined from dose-response curves obtained after subjecting each agent to the tail suspension test.

Another dose-response curve was subsequently obtained and analysed upon coadministration of antidepressants with XAE or with XA in a fixed-ratio (1 : 1) combination based on fractions 1/2, 1/4, and 1/8 of their respective ED_50_s as obtained from the tail suspension test.

An isobologram was then constructed by connecting the plot of the theoretical ED_50_s of the antidepressants (plotted on the ordinate) with those of the XAE or XA (plotted on the abscissa) so as to attain an additivity line. The experimental ED_50_ and its associated 95% confidence interval for each drug mixture were determined by linear regression analysis of the log dose-response curve and compared by a *t*-test to a theoretical additive ED_50_ obtained via calculation using the formula:

*Z*_add_ = *f* (ED_50_) of antidepressant + (1 − *f*) (ED_50_) of extract or isolate, where *f* is the fraction of each component in the mixture and the variance (Var) of *Z*_add_ was also calculated using the following formula:

Var*Z*_add_ = *f*^2^ (Var ED_50_ of antidepressant) + (1 − *f*)^2^ ED_50_ of extract or isolate.

The S.E.M.s were determined from the variances and resolved based on the ratio of the individual drugs present in each combination.

### 2.7. Plasma Concentration

Mice were well-fed prior to the experiment, and they were provided water *ad libitum*. The mice (*n* = 5) were grouped and given a single oral dose of ED_50_ of each antidepressant and xylopic acid, a combination of ED_50_ of antidepressant and XA/XAE, and 10 mLkg^−1^ of vehicle as control.

Blood samples were subsequently collected via jugular venepuncture at predetermined time points of 0, 6, 12, 18, and 24 h. The blood samples collected were processed and analysed via HPLC using a method previously described [21]. The column employed was Phenomenex Luna 3*µ* C18 (2) 100A 150 × 4.60 mm 3 micron. The mobile phase contained 0.05% trifluoroacetic acid (10%) and methanol (90%). The eluent was monitored at 206 nm.

### 2.8. Data Analysis

The data were presented as mean ± S.E.M. The dose-response curves plotted were subjected to a two-way (treatment x time) repeated-measure analysis of variance (ANOVA) followed by Tukey's multiple comparisons test. An iterative nonlinear regression (3-parameter logistic) equation was used in the determining ED_50_ of all agents alone and in their various combinations from the respective dose-response curves.

(1) $$\begin{array}{c} Y=a+\frac{b-a}{1+10^{logED50-x}} \end{array}$$

where *X* is the logarithm of dose and *Y* is the response. *Y* starts at *a* (bottom) and goes to *b* (top) with a sigmoid shape. The fitted midpoints (ED_50_s) of the curves were compared statistically using the *F* test. Microsoft® Excel® and GraphPad Prism for Windows version 8 (GraphPad Software, San Diego, CA, USA) were used in performing isobolographic calculations, and the corresponding isoboles were subsequently plotted using GraphPad Prism for Windows version 8 (GraphPad Software, San Diego, CA, USA).

Noncompartmental analysis was employed in the monitoring of drug plasma concentration. Statistical difference between AUC of antidepressants alone and AUC of antidepressants after the administration of combination treatment was determined by Dunnett's T3 multiple comparison test using GraphPad Prism for Windows version 8 (GraphPad Software, San Diego, CA, USA).

## 3. Results

### 3.1. HPLC Analysis

**Figure 1:**
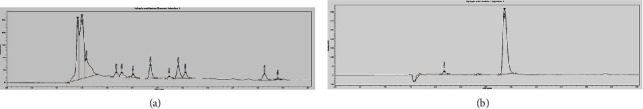
HPLC-UV chromatogram of *Xylopia aethiopica* extract in methanol using isocratic elution of methanol : water (90 : 10). Detection wavelength = 206 nm (a). HPLC-UV chromatogram of isolated xylopic acid in methanol using isocratic elution of methanol : water (90 : 10). Detection wavelength = 206 nm. *R*_*t*_ = 3.368 min (b).

The HPLC-UV fingerprinting of *Xylopia aethiopica* extract produced a chromatogram, which showed twelve peaks with varying retention times, which was indicative of twelve different components being present in the *Xylopia aethiopica* extract (Figure 1). The isolated xylopic acid had a fairly prominent peak, indicative of a very pure isolate. The HPLC retention time of isolated xylopic acid was 3.368 (Figure 1).

### 3.2. Determination of ED_50_ in Tail Suspension Test

**Figure 2:**
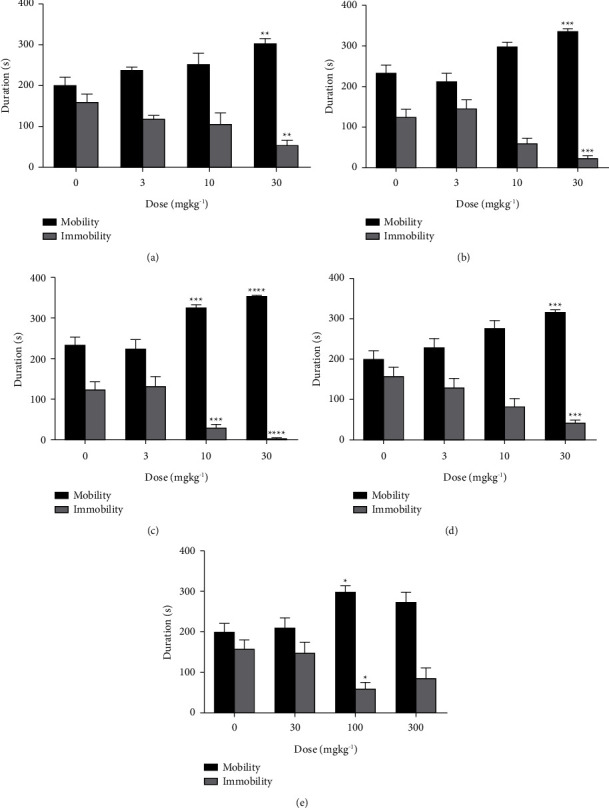
Antidepressant effect of imipramine (3–30 mgkg^−1^) (a), fluoxetine (3–30 mgkg^−1^) (b), venlafaxine (3–30 mgkg^−1^) (c), xylopic acid (3–30 mgkg^−1^) (d), and *Xylopia aethiopica* extract (30–300 mgkg^−1^) (e) in tail-suspended mice. Black shaded bars represent mobility time, while grey shaded bars represent immobility time. ^*∗*^*P* < 0.05, ^*∗∗*^*P* < 0.01, ^*∗∗∗*^*P* < 0.001, ^*∗∗∗∗*^*P* < 0.0001 (two-way ANOVA). Data points are mean ± S.E.M. of *n* = 7 mice. Fluoxetine (FL), venlafaxine (VL), xylopic acid (XA), and *Xylopia aethiopica* extract (XAE).

**Figure 3:**
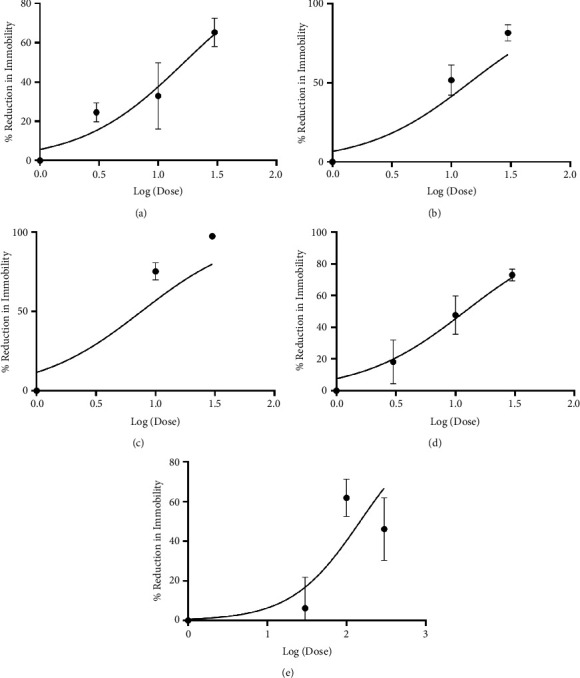
Dose-response curve of imipramine (3–30 mgkg^−1^) (a), fluoxetine (3–30 mgkg^−1^) (b), venlafaxine (3–30 mgkg^−1^) (c), xylopic acid (3–30 mgkg^−1^) (d), and *Xylopia aethiopica* extract (30–300 mgkg^−1^) (e) in tail-suspended mice. Data points are mean ± S.E.M. of *n* = 7 mice. Fluoxetine (FL), venlafaxine (VL), xylopic acid (XA), and *Xylopia aethiopica* extract (XAE).

Xylopic acid, *Xylopia aethiopica* extract, fluoxetine, and venlafaxine significantly reduced immobility time while correspondingly increasing mobility time in the test mice (Figure 2). The effects observed were predominantly dose-dependent, with the highest dose resulting in the highest effect. XA, XAE, FL, VL, and IP produced peak effects of 72.99% (F_3,46_ = 16.62, *P* < 0.0001; Figure 3(d)), 61.89% (F_3,44_ = 10.61, *P* < 0.0001; Figure 3(e)), 81.49% (F_3,48_ = 26.98, *P* < 0.0001; Figure 3(b)), 97.43% (F_3,46_ = 39.76, *P* < 0.0001; Figure 3(c)), and 65.32% (F_3,48_ = 11.04, *P* < 0.0001; Figure 3(a)), respectively. Venlafaxine was the most potent (ED_50_: 7.60 ± 2.42 mgkg^−1^; Table 1; Figure 3) of all the agents, followed by xylopic acid (ED_50_: 12.04 ± 2.92 mgkg^−1^; Table 1; Figure 3), fluoxetine (ED_50_: 14.21 ± 4.69 mgkg^−1^; Table 1; Figure 3), imipramine (ED_50_: 16.57 ± 4.29 mgkg^−1^; Table 1; Figure 3), and then the extract (ED_50_: 148.60 ± 51.98 mgkg^−1^; Table 1; Figure 3).

### 3.3. Isobologram of Drug Combinations

**Figure 4:**
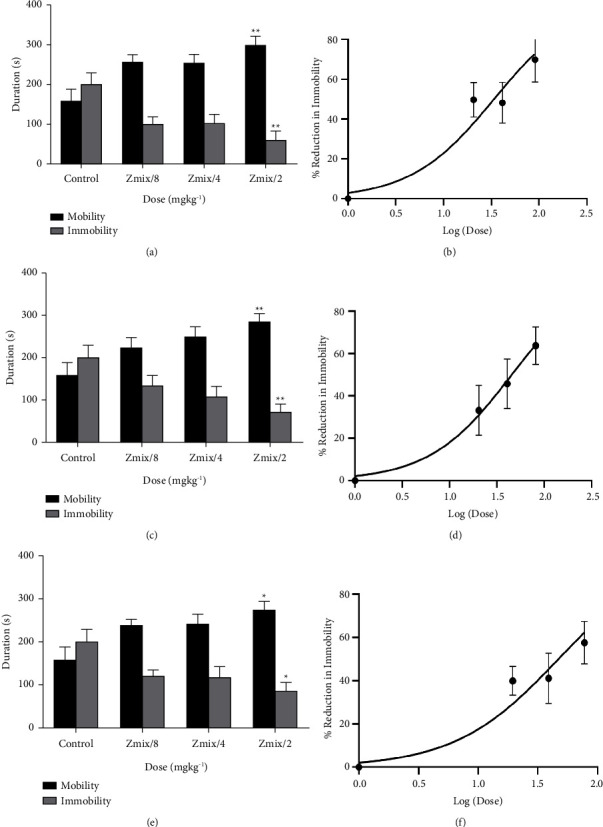
Antidepressant effect of coadministration of *Xylopia aethiopica* extract with (a, b) imipramine, (c, d) fluoxetine, (e, f) venlafaxine. ^*∗*^*P* < 0.05, ^*∗∗*^*P* < 0.01, ^*∗∗∗*^*P* < 0.001, ^*∗∗∗∗*^*P* < 0.0001 (ANOVA). Each point represents the mean ± S.E.M. (*n* = 7).

The combination of *Xylopia aethiopica* extract with imipramine, fluoxetine, and venlafaxine all showed significant (XAE/IP: F_(3,44)_ = 13.26, *P* < 0.0001; XAE/FL: F_(3,44)_ = 10.30, *P* < 0.0001; XAE/VL: F_(3,46)_ = 9.65, *P* < 0.0001) antidepressant activity, evidenced by their ability to reduce immobility time and consequently increase escape-oriented activity (mobility time) in tail-suspended mice (Figure 4). The peak effect of 69.87% was observed at *Z*_mix_/2 of the imipramine-extract combination.

**Figure 5:**
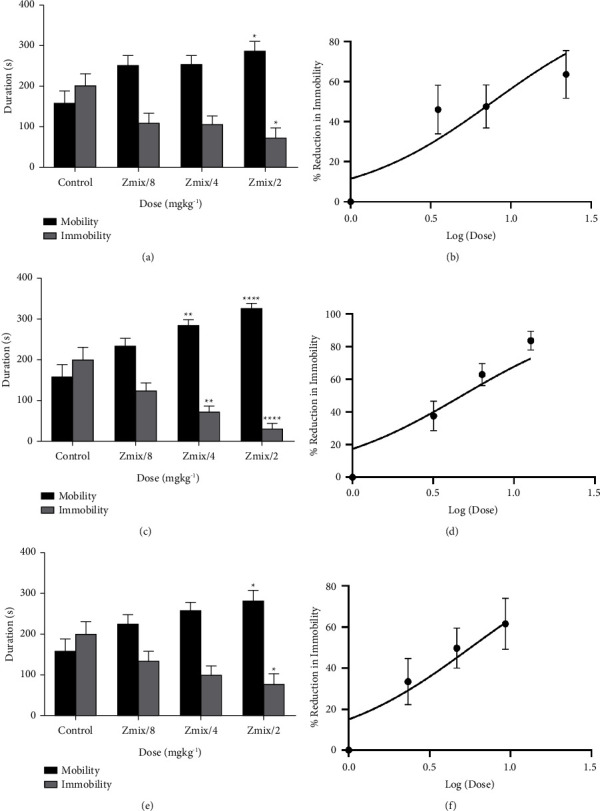
Antidepressant effect of coadministration of xylopic acid with (a, b) imipramine, (c, d) fluoxetine, (e, f) venlafaxine. ^*∗*^*P* < 0.05, ^*∗∗*^*P* < 0.01, ^*∗∗∗*^*P* < 0.001, ^*∗∗∗∗*^*P* < 0.0001 (ANOVA). Each point represents the mean ± S.E.M. (*n* = 7).

With xylopic acid, combinations with imipramine, fluoxetine, and venlafaxine also resulted in significant (XA/IP: F_(3,46)_ = 9.27, *P* < 0.0001; XA/FL: F_(3,42)_ = 26.74, *P* < 0.0001; XA/VL: F_(3,46)_ = 9.45, *P* < 0.0001) antidepressant activity. The duration-dose chart showed a significant reduction in immobility time with a correspondingly significant increase in mobility time in tail-suspended mice (Figure 5). The peak effect of 83.72% was observed at *Z*_mix_/2 of the fluoxetine-xylopic acid combination.

#### 3.3.1. Isobologram of *Xylopia aethiopica* Extract with Antidepressants

**Figure 6:**
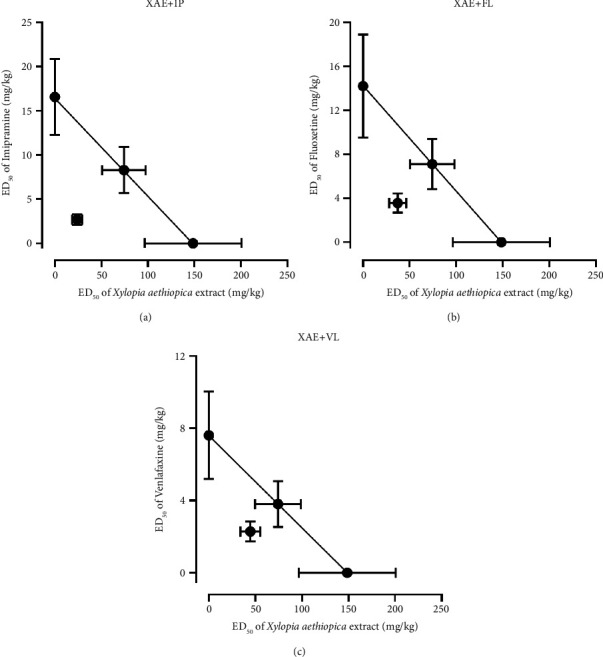
Isobolograms for oral coadministration of *Xylopia aethiopica* extract with (a) imipramine, (b) fluoxetine, (c) venlafaxine in the tail suspension test. The theoretical ED_50_ for an additive effect and the experimental ED_50_ values are represented in the graphs.

For combinations involving *Xylopia aethiopica* extract, the theoretical ED_50_ (*Z*_add_) for combinations with fluoxetine, venlafaxine, and imipramine was 81.41 ± 26.10 mgkg^−1^, 78.10 ± 26.02 mgkg^−1^, and 82.59 ± 26.08 mgkg^−1^, respectively (Table 2). The experimental ED_50_ (*Z*_mix_) of combinations of extract with fluoxetine, venlafaxine, and imipramine was determined to be 40.83 ± 10.03 mgkg^−1^, 46.90 ± 11.18 mgkg^−1^, and 26.62 ± 5.98 mgkg^−1^, respectively (Table 2). A Student's “*t*” test comparing the theoretical and experimental ED_50_s showed that *Z*_mix_ was significantly less than *Z*_add_ for all combinations (FL/XAE: *P*=0.0024; VL/XAE: *P*=0.0130; IP/XAE: *P*=0.0001) (Table 2; Figure 6).

#### 3.3.2. Isobologram of Xylopic Acid with Antidepressants

**Figure 7:**
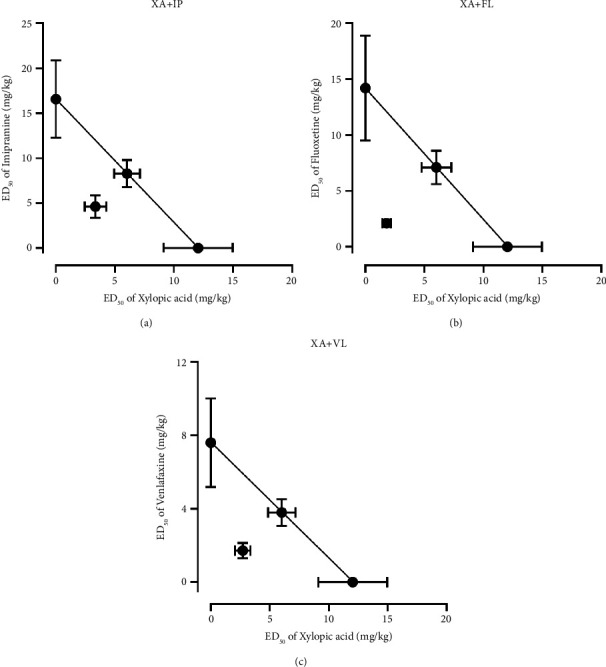
Isobolograms for oral coadministration of xylopic acid with (a) imipramine, (b) fluoxetine, and (c) venlafaxine. The theoretical ED_50_ for an additive effect and the experimental ED_50_ values are represented in the graphs.

With regard to the isolate, xylopic acid, the theoretical additive ED_50_ (*Z*_add_) of the combinations with fluoxetine, venlafaxine, and imipramine was computed to be 13.13 ± 2.76 mgkg^−1^, 9.82 ± 1.90 mgkg^−1^, and 14.31 ± 2.59 mgkg^−1^, respectively (Table 3). The corresponding experimental ED_50_ (*Z*_mix_) for combinations with fluoxetine, venlafaxine, and imipramine was determined to be 3.91 ± 0.78 mgkg^−1^, 4.43 ± 1.08 mgkg^−1^, and 7.95 ± 2.17 mgkg^−1^, respectively (Table 3). A Student's “*t*” test comparing the theoretical and experimental ED_50_s showed that *Z*_mix_ was significantly less than *Z*_add_ in all combinations (FL/XA: *P* < 0.0001; IP/XA: *P*=0.0003; and VL/XA: *P* < 0.0001) (Table 3; Figure 7).

### 3.4. Plasma Concentration

Upon monitoring the plasma concentration of antidepressants and xylopic acid after administration of drug combinations to test mice via noncompartmental analysis, it was observed that *Xylopia aethiopica* extract and xylopic acid significantly altered the amount of imipramine in systemic circulation, and venlafaxine and fluoxetine significantly altered the amount of xylopic acid in systemic circulation. The other combinations, however, did not cause any significant changes in the amount of antidepressants or xylopic acid getting into systemic circulation.

**Figure 8:**
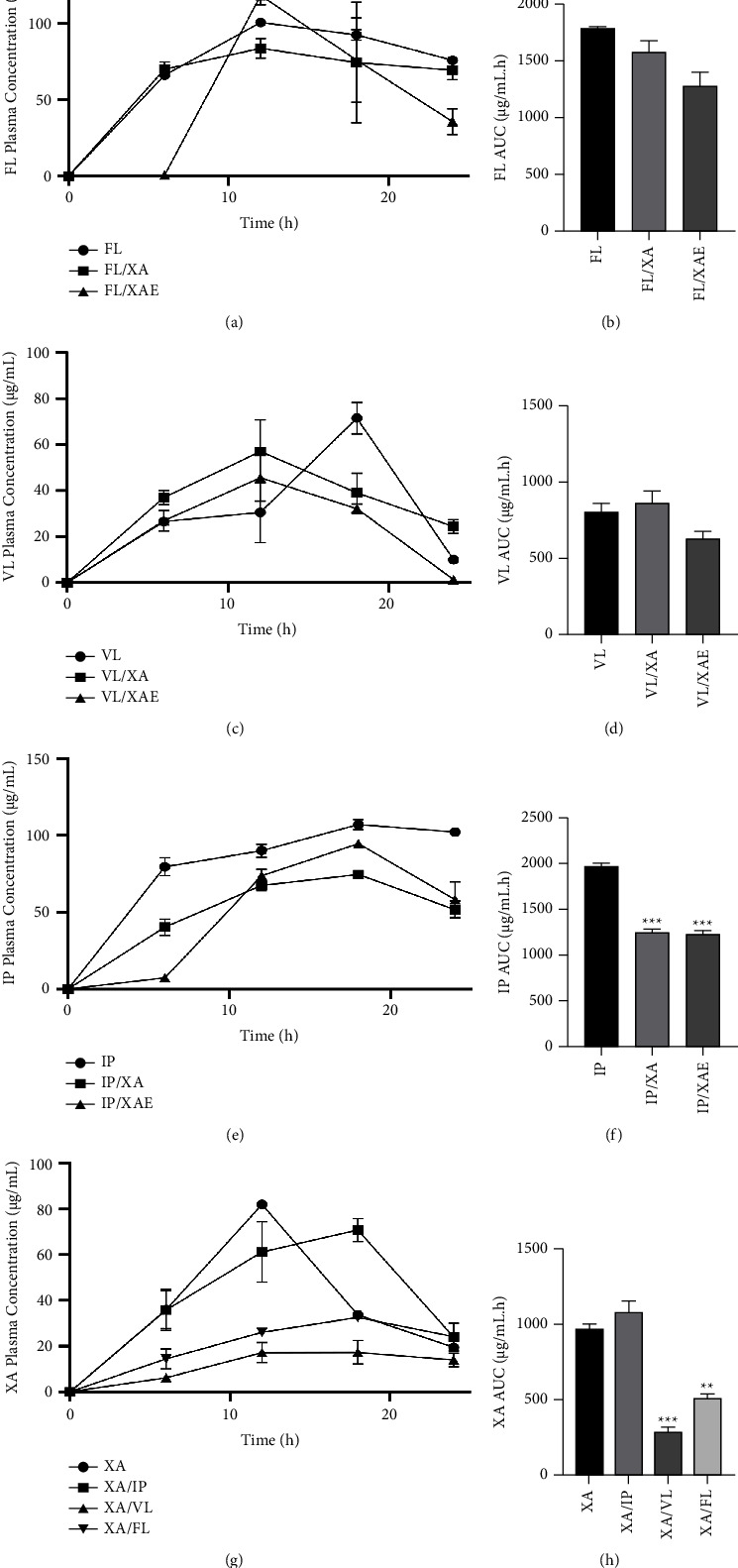
Concentration-time plot of various agents and their combinations after oral administration of their ED_50_s and their corresponding areas under the curve. ^*∗*^*P* < 0.05, ^*∗∗*^*P* < 0.01, ^*∗∗∗*^*P* < 0.001, ^*∗∗∗∗*^*P* < 0.0001 (one-way ANOVA). Each point represents the mean ± S.E.M. (*n* = 5).

*Xylopia aethiopica* extract and xylopic acid caused a significant reduction in the AUC of imipramine from 1966 ± 58.98 *µ*g/ml.h to 1228 ± 67.40 *µ*g/ml.h (*P* < 0.0001) and 1250 ± 55.95 *µ*g/ml.h (*P* < 0.0001), respectively (Figure 8). *Xylopia aethiopica* extract and xylopic acid were also able to cause a decline in *C*_max_ from 107.07 ± 5.55 mgkg^−1^ to 94.55 ± 0.75 mgkg^−1^ and 74.59 ± 1.74 mgkg^−1^, respectively (Table 4). The extract reduced the half-life of imipramine from 44.38 h to 18.22 h, but the isolate, however, increased the half-life of imipramine from 44.38 h to 50.48 h (Table 4). The extract and isolate did not significantly alter the AUC of fluoxetine (Table 5) and venlafaxine (Table 6).

Venlafaxine and fluoxetine negatively altered the AUC of xylopic acid, resulting in a decline from 968.10 ± 61.22 *µ*g/ml.h to 285.90 ± 51.92 *µ*g/ml.h (*P* < 0.0001) and 510.60 ± 44.74 *µ*g/ml.h (*P* < 0.005), respectively (Table 7; Figure 8). *C*_max_ of xylopic acid was also reduced from 81.94 ± 0.73 mgkg^−1^ to 17.30 ± 8.92 mgkg^−1^ when coadministered with venlafaxine and 32.53 ± 1.58 mgkg^−1^ when coadministered with fluoxetine (Table 7). The half-life of xylopic acid was also altered by venlafaxine and fluoxetine from 5.78 h to 21.31 h and 26.63 h, respectively (Table 7).

## 4. Discussion

Depression is a psychiatric disorder affecting an estimated 350 million people worldwide and is implicated in about 60% of suicides [7, 22]. Despite the strides made in the treatment of depression, up to 30% of patients do not meet therapeutic goals due to varying reasons, including drug interaction with other drugs or herbs [12, 15–17].

This study investigated potential herb-drug interactions that might occur between antidepressants and the hydroethanolic extract of the popularly used herb, *Xylopia aethiopica*, or its isolate, xylopic acid. The study found a synergistic pharmacodynamic herb-drug interaction between the extract and all the standard antidepressants, and between the isolate and all the standard antidepressants. The pharmacokinetic dispositions of imipramine and xylopic acid were altered.

*Xylopia aethiopica* extract, xylopic acid, and antidepressants (imipramine, fluoxetine, and venlafaxine) all showed significant antidepressant-like and antidepressant activity when subjected to the tail suspension test—a model for detecting potential antidepressants [23]. All the agents elongated the duration spent by tail-suspended mice engaging in escape-oriented behaviour (pedalling, curling, and swinging), while correspondingly reducing the duration spent being immobile.

This antidepressant-like activity of *Xylopia aethiopica* extract has been previously established to be due to the ability of the extract to interact with serotonergic neurotransmission, with a possible glutamatergic effect via glycine_B_ co-binding site and nitric oxide synthase inhibition [3]. Additionally, the antidepressant-like activity of the extract was likely due to the presence of some secondary metabolites (sterols, flavonoids, and xylopic acid), which are known to possess antidepressant-like properties [20, 24, 25]. UV fingerprinting of the extract, as shown in this study, revealed the presence of several components. Similarly, xylopic acid owes its antidepressant-like activity to its effect on serotonergic mechanisms, as well as neuroprotective mechanisms, involving brain-derived neurotrophic factor (BDNF) and antioxidant enzymes [20]. The exact mechanisms by which the extract and isolate affect serotonergic neurotransmission, however, remain unknown. The standard antidepressants inhibit the reuptake of neurotransmitters, resulting in elevated levels of these neurotransmitters. Imipramine inhibits the reuptake of serotonin, dopamine, and noradrenaline; fluoxetine inhibits the reuptake of serotonin; and venlafaxine inhibits the reuptake of serotonin and noradrenaline [11].

Isobolographic analysis of drug combinations of *Xylopia aethiopica* extract with antidepressants and xylopic acid with antidepressants revealed that the experimental ED_50_s were significantly less than the theoretical additive ED_50_s for all the drug combinations studied. These were indicative of synergistic pharmacodynamic interactions due to increased potency [26–28]. These synergistic interactions serve as a tool to examine drug mechanisms, as the synergistic effects observed suggest that the agents in each combination either act simultaneously at distinct sites or activate different pathways [29].

It can therefore be inferred that both *Xylopia aethiopica* extract and xylopic acid potentiated the antidepressant activities of imipramine, fluoxetine, and venlafaxine, possibly by elevating serotonergic neurotransmission via mechanisms that vary from those employed by the antidepressants. The observed synergistic effects could have also been contributed by the other nonserotonergic mechanisms of *Xylopia aethiopica* extract and xylopic acid as established in previous studies [3, 20].

Pharmacokinetically, *Xylopia aethiopica* extract and xylopic acid interfered with the oral absorption of imipramine, resulting in a significant reduction in the amount of imipramine the body of the mice was exposed to. A previous study found that *Xylopia aethiopica* extract had smooth muscle relaxant properties via the serotonergic pathway, hence hindering gastric emptying [30].

Gastric emptying is an important factor that affects drug absorption and is usually the rate-limiting step in the absorption of xenobiotics, as it defines how quickly a xenobiotic gets to the upper small intestine, where absorption is greatest [30, 31].

By impeding gastric emptying, *Xylopia aethiopica* extract was able to cause a decline in the amount of imipramine to get into systemic circulation. Previous studies have shown that *Xylopia aethiopica* extract and xylopic acid share a similarity in their mechanism of action via the serotonergic pathway [3, 20]. Hence, xylopic acid is likely to have a similar effect on gastric emptying as *Xylopia aethiopica* extract, resulting in the reduced amount of imipramine getting into systemic circulation that was observed. Venlafaxine and fluoxetine also reduced the amount of xylopic acid getting into systemic circulation after oral administration by possibly slowing down gastric emptying [32, 33].

Xylopic acid also decreased the elimination rate of imipramine due to its substrate activity on p-glycoprotein, hence competing with imipramine—which is also a substrate of p-glycoprotein—for binding site [34–37]. This resulted in the inefficient efflux of imipramine from systemic circulation and therefore slowing down the elimination process [38]. Venlafaxine and fluoxetine also likely had a similar effect on xylopic acid as they are both substrates of p-glycoprotein [35, 36].

The inhibitory effect of xylopic acid on CYP 3A4 could have contributed to the decrease in the elimination rate of imipramine, as the enzyme is responsible for metabolising imipramine [34, 39, 40]. Venlafaxine and fluoxetine possibly had a similar effect on xylopic acid as they are also potent inhibitors of CYP 2D6, a metaboliser of xylopic acid, hence, decreasing the elimination rate of xylopic acid [34, 39, 41, 42].

With *Xylopia aethiopica* extract and xylopic acid both having the ability to alter the pharmacodynamic and pharmacokinetic disposition of antidepressant, this could impact the clinical management of depression in patients who coadminister the extract or isolate with their antidepressants. Herb-drug coadministration could improve clinical therapeutic outcomes, as the potential elevated antidepressant activity made possible due to the synergistic relationship between herb and antidepressant would provide a greater resolution of depression as compared to the administration of antidepressants alone. Prescribers might therefore encourage coadministration of the extract or isolate with antidepressants. The dose of imipramine being administered may need to be adjusted higher as the amount of imipramine getting into systemic circulation could be significantly reduced by either the extract or isolate. This may require the plasma concentration of imipramine in patients to be constantly monitored to ensure that therapeutic range and steady-state concentration of imipramine are attained.

## 5. Conclusion

*Xylopia aethiopica* extract and xylopic acid potentiated the antidepressant activity of imipramine, venlafaxine, and fluoxetine, possibly by elevating serotonergic neurotransmission via varying [6] pathways.

Xylopic acid and *Xylopia aethiopica* extract reduced the amount of imipramine in systemic circulation, and venlafaxine and fluoxetine reduced the amount of xylopic acid in systemic circulation, likely via decreasing the rate of gastric emptying.

### 5.1. Study Limitations

In assessing potential sources of bias or confounding factors, efforts to minimise bias through blinding techniques during behavioural assessments were carried out. With regard to external validity of the study's findings, the limitations inherent in animal models should be acknowledged. These models, while valuable, might restrict direct extrapolation to human responses due to variances in pharmacokinetics, physiology, and behavioural nuances between species. Furthermore, while the chosen antidepressants mirror major clinical classes, the exclusive focus on these agents might overlook differences in response patterns with other antidepressants when combined with the herbal extract or isolated compound under investigation. These factors collectively highlight the necessity for cautious interpretation and the potential limitations in directly applying findings from this animal study to clinical scenarios in human populations.

## Figures and Tables

**Table 1:** The potencies and peak effects of the various agents used in the tail suspension test.

Drugs	ED_50_ (mgkg^−1^)	*E*_max_ (%)
Xylopic acid	12.04 ± 2.92	72.99
Fluoxetine	14.21 ± 4.69	81.49
Venlafaxine	7.60 ± 2.42	97.43
Imipramine	16.57 ± 4.29	65.32
*Xylopia aethiopica* extract	148.60 ± 51.98	61.89

**Table 2:** Theoretical and experimental ED_50_ ± S.E.M. of *Xylopia aethiopica* extract and antidepressant combinations in the tail suspension test and the corresponding computed indices.

Combinations	XAE/FL	XAE/VL	XAE/IP
Theoretical ED_50_ (mgkg^−1^)	81.41 ± 26.10	78.10 ± 26.02	82.59 ± 26.08
Experimental ED_50_ (mgkg^−1^)	40.83 ± 10.03^*∗∗*^	46.90 ± 11.18^*∗*^	26.62 ± 5.98^*∗∗∗*^
Interaction index (*γ*)	0.502	0.601	0.322
Drugs ratio	10.457 : 1	19.553 : 1	8.968 : 1

^*∗*^ *P* ≤ 0.05, ^*∗∗*^*P* < 0.01, and ^*∗∗∗*^*P* < 0.001 (Student's “*t*” test) compared experimental ED_50_ to theoretical ED_50_. Values are expressed as mean ± S.E.M.

**Table 3:** Theoretical and experimental ED_50_ ± S.E.M. of xylopic acid and antidepressant combinations in the tail suspension test and the corresponding computed indices.

Combinations	XA/FL	XA/VL	XA/IP
Theoretical ED_50_ (mgkg^−1^)	13.13 ± 2.76	9.82 ± 1.90	14.31 ± 2.59
Experimental ED_50_ (mgkg^−1^)	3.91 ± 0.78^*∗∗∗∗*^	4.43 ± 1.08^*∗∗∗∗*^	7.95 ± 2.17^*∗∗∗*^
Interaction index (*γ*)	0.298	0.451	0.556
Drugs ratio	0.847 : 1	1.584 : 1	0.727 : 1

^*∗*^ *P* ≤ 0.05, ^*∗∗∗*^*P* < 0.001, and ^*∗∗∗∗*^*P* < 0.0001 (Student's “*t*” test) compared experimental ED_50_ to theoretical ED_50_. Values are expressed as mean ± S.E.M.

**Table 4:** Effect of *Xylopia aethiopica* extract and xylopic acid on the pharmacokinetic disposition of imipramine.

Parameter	Unit	IP	IP/XA	IP/XAE
*t*_1/2_	h	44.38	50.48	18.22
*C*_max_	*µ*g/ml	107.07 ± 5.55	74.59 ± 1.74	94.55 ± 0.75
*T*_max_	h	18	18	18
AUC_*o*-*t*_	*µ*g/ml.h	1966 ± 58.98	1250 ± 55.95^*∗∗∗*^	1228 ± 67.40^*∗∗∗*^

^*∗*^ *P* ≤ 0.05, ^*∗∗∗*^*P* < 0.001 (Dunnett's T3 multiple comparisons test) compared AUC of coadministrations to AUC of single-agent administration. Values are expressed as mean ± S.E.M (*n* = 5).

**Table 5:** Effect of *Xylopia aethiopica* extract and xylopic acid on the pharmacokinetic disposition of fluoxetine.

Parameter	Unit	FL	FL/XA	FL/XAE
*t*_1/2_	h	29.45	44.36	6.95
*C*_max_	*µ*g/ml	100.71 ± 3.10	83.66 ± 6.39	118.33 ± 10.74
*T*_max_	h	12	12	12
AUC_*o*-*t*_	*µ*g/ml.h	1783 ± 33.00	1577 ± 171.70	1278 ± 212.40

^*∗*^ *P* ≤ 0.05 (Dunnett's T3 multiple comparisons test) compared AUC of coadministrations to AUC of single-agent administration. Values are expressed as mean ± S.E.M (*n* = 5).

**Table 6:** Effect of *Xylopia aethiopica* extract and xylopic acid on the pharmacokinetic disposition of venlafaxine.

Parameter	Unit	VL	VL/XA	VL/XAE
*t*_1/2_	h	7.39	9.80	2.32
*C*_max_	*µ*g/ml	71.43 ± 9.57	56.94 ± 23.88	45.54 ± 17.41
*T*_max_	h	18	12	12
AUC_*o*–*t*_	*µ*g/ml.h	801.20 ± 105.40	870.70 ± 121.90	631.00 ± 82.14

^*∗*^ *P* ≤ 0.05 (Dunnett's T3 multiple comparisons test) compared AUC of coadministrations to AUC of single-agent administration. Values are expressed as mean ± S.E.M (*n* = 5).

**Table 7:** Effect of antidepressants on the pharmacokinetic disposition of xylopic acid.

Parameter	Unit	XA	IP/XA	VL/XA	FL/XA
*t*_1/2_	h	5.78	5.21	21.31	26.63
*C*_max_	*µ*g/ml	81.94 ± 0.73	70.80 ± 8.84	17.30 ± 8.92	32.53 ± 1.58
*T*_max_	h	12	18	18	18
AUC_*o*-*t*_	*µ*g/ml.h	968.10 ± 61.22	1079.00 ± 127.60	285.90 ± 51.92^*∗∗∗*^	510.60 ± 44.74^*∗∗*^

^*∗*^ *P* ≤ 0.05, ^*∗∗*^*P* < 0.01, and ^*∗∗∗*^*P* < 0.001 (Dunnett's T3 multiple comparisons test) compared AUC of coadministrations to AUC of single-agent administration. Values are expressed as mean ± S.E.M (*n* = 5).

## Data Availability

The data used to support the findings of this study are available from the corresponding author upon request.
